# Variations in the Antivirulence Effects of Fatty Acids and Virstatin against *Vibrio cholerae* Strains

**DOI:** 10.4014/jmb.2405.05002

**Published:** 2024-07-19

**Authors:** Donghyun Lee, Jayun Joo, Hunseok Choi, Seonghyeon Son, Jonghyun Bae, Dong Wook Kim, Eun Jin Kim

**Affiliations:** 1 Department of Pharmacy, College of Pharmacy, Hanyang University, Ansan 15588, Republic of Korea; 2 Institute of Pharmacological Research, Hanyang University, Ansan 15588, Republic of Korea

**Keywords:** Cholera, *Vibrio cholerae*, cholera toxin (CT), toxin co-regulated pilus (TCP), ToxT, fatty acid, virstatin

## Abstract

The expression of two major virulence factors of *Vibrio cholerae*, cholera toxin (CT) and toxin co-regulated pilus (TCP), is induced by environmental stimuli through a cascade of interactions among regulatory proteins known as the ToxR regulon when the bacteria reach the human small intestine. ToxT is produced via the ToxR regulon and acts as the direct transcriptional activator of CT (*ctxAB*), TCP (*tcp* gene cluster), and other virulence genes. Unsaturated fatty acids (UFAs) and several small-molecule inhibitors of ToxT have been developed as antivirulence agents against *V. cholerae*. This study reports the inhibitory effects of fatty acids and virstatin (a small-molecule inhibitor of ToxT) on the transcriptional activation functions of ToxT in isogenic derivatives of *V. cholerae* strains containing various *toxT* alleles. The fatty acids and virstatin had discrete effects depending on the ToxT allele (different by 2 amino acids), *V. cholerae* strain, and culture conditions, indicating that *V. cholerae* strains could overcome the effects of UFAs and small-molecule inhibitors by acquiring point mutations in *toxT*. Our results suggest that small-molecule inhibitors should be examined thoroughly against various *V. cholerae* strains and *toxT* alleles during development.

## Introduction

*Vibrio cholerae*, a gram-negative bacterium, causes cholera, a severe diarrheal disease, in humans [1, 2]. The disease is triggered by the secreted cholera toxin (CT), which is translocated into intestinal epithelial cells and increases the cyclic AMP concentration in host cells [3]. *V. cholerae* strains that belong to two serogroups, O1 and O139, produce CT and cause epidemic cholera, whereas strains belonging to other serogroups—non-O1, non-O139—can cause sporadic gastroenteritis [3].

The O1 serogroup strains are further divided into two biotypes, classical and El Tor, using biochemical and microbiological characteristics [4]. In addition to biotype-specific characteristics that result from genomic differences, strains belonging to the two biotypes usually produce biotype-specific CT [4, 5]. The active subunit (CTA) is the same in all the biotype-specific CTs, and two amino acids in the binding subunit (CTB) differ (His and Thr at the 39^th^ and 68^th^ amino acid positions in classical biotype strains and Tyr and Ile in El Tor biotype strains) [6]. Nonetheless, atypical El Tor biotype strains that produce classical biotype CTB have been prevalent globally since the early 1990s [6-8].

Another virulence factor important for provoking disease symptoms, the toxin co-regulated pilus (TCP), is also produced by *V. cholerae* [9]. TCP is a type IV pilus that contains the subunit protein TcpA [10]. TCP facilitates microcolony formation during colonization of the human small intestine and also acts as the receptor for CTXΦ, which carries CT genes (*ctxAB*) [11, 12].

In the human small intestine, environmental stimuli initiate the expression of *V. cholerae* virulence genes through a coordinated cascade of regulatory proteins called the ToxR regulon [13, 14]. TcpP and TcpH are produced by environmental stimuli and form a complex with ToxR and ToxS that induces the production of ToxT—an AraC/XylS-type transcriptional regulator [14, 15]. ToxT directly activates the transcription of CT and TCP [16, 17].

The detailed mechanisms of the ToxR regulon and virulence gene expression in *V. cholerae* have been principally obtained by *in vitro* studies using virulence-inducing culture conditions. Various virulence-inducing culture conditions have been developed, including agglutinating culture conditions (bacterial culture at 30°C in LB medium at pH 6.5) for classical biotype strains [18] and AKI conditions [incubation in AKI broth (1.5% Bacto peptone, 0.4% yeast extract, 0.5% NaCl, 0.3% NaHCO_3_) at 37°C for 4 hr in static conditions followed by vigorous shaking for 16 h] for El Tor biotype strains [19, 20]. Although many *V. cholerae* strains of each biotype express virulence genes in biotype-specific virulence gene-inducing culture conditions, there are exceptional strains that do not [21, 22]. However, virulence gene expression in *V. cholerae* can be greatly increased in simpler culture conditions by introducing alternative *toxT* alleles [22-24]. While biotype-specific virulence gene-inducing culture conditions are required for expression of virulence genes in many *V. cholerae* strains that contained the *toxT*-SY allele (Ser and Tyr at the 65^th^ and 139^th^ amino acid positions, respectively), replacement of the *toxT*-SY allele with *toxT*-SF (Ser and Phe at the 65^th^ and 139^th^ amino acid positions) allele facilitated the virulence gene expression in simple laboratory culture conditions [22-24]. Further detailed virulence gene-inducing functions of alternative *toxT* alleles are described below.

Unsaturated fatty acids (UFAs) and several small-molecule inhibitors of ToxT have been developed as potential antivirulence agents to reduce the expression of virulence genes following *V. cholerae* infection [25-27]. Several UFAs, which are the components of bile, have been shown to inhibit the expression of virulence genes by ToxT [28] and thus have been used to develop a potential treatment for cholera [29]. A study of the full-length ToxT structure revealed that *cis*-palmitoleate prevents ToxT from binding to DNA [30]. Moreover, small-molecular inhibitors of ToxT have been developed to effectively reduce the virulence of *V. cholerae* [25-27], and one of them, virstatin, has been shown to reduce ToxT dimerization and thereby diminish virulence gene expression [31]. Additionally, studies of the ToxT structure in the absence of UFAs or an inhibitor showed that free ToxT is conformationally flexible for dimerization and binding to DNA [32].

For this study, we investigated the inhibitory effects of fatty acids and virstatin on the transcriptional activator functions of ToxT using isogenic derivatives of *V. cholerae* strains containing various *toxT* alleles. Two UFAs (linoleic acid and oleic acid) and two saturated fatty acids (SFAs, palmitic acid and stearic acid), all abundant fatty acid components of bile, were examined [28].

Virulence gene expression in the tested *V. cholerae* strains was inhibited by UFAs and virstatin; however, those inhibitory effects were weakened by alternative *toxT* alleles and culture conditions. SFAs had previously been shown to have no significant effects on the transcriptional activator functions of ToxT, but our results indicate that they can interfere with ToxT activities depending on the strain, *toxT* allele, and culture conditions. Our results suggest that *V. cholerae* could develop resistance against fatty acids or small-molecular inhibitors by accumulating point mutations in *toxT*.

## Materials and Methods

### Bacterial Strains

The bacterial strains used in this study are listed in Table 1.

### *toxT* Allele Exchange in *V. cholerae* Strains

The construction of isogenic derivatives of each *V. cholerae* strains that contained variant *toxT* allele have been described previously [21-24]. Briefly, four *toxT* alleles were individually inserted into a suicidal plasmid, pCVD442, then conjugally transferred to each strain to construct isogenic derivatives by allelic exchange methods. Excision of the suicidal vector was screened on sucrose-containing LB agar plate. The replacement of the original *toxT* allele by alternative alleles within the *toxT* ORF in isogenic derivatives of each *V. cholerae* strain was confirmed by Sanger sequencing.

### Bacterial Culture

**Culture in LB medium.** *V. cholerae* strains were maintained on LB agar plates containing streptomycin (100 μg/ml). A single colony was inoculated in 3 ml of LB medium and cultured overnight at 30°C. Approximately 100 μl of the overnight cultured bacteria were used to inoculate 10 ml of LB medium containing 0.02% fatty acid or 50 μM virstatin and cultured for 18 h at either 30°C or 37°C [47]. PA, LA, and OA were prepared as 2% stock solutions in methanol, and SA was prepared as a 2% stock solution in ethanol. Therefore, 100 μl of methanol or ethanol was added as a negative control culture for the fatty acids. The virstatin stock solution was prepared at a concentration of 25 mM in DMSO, and 20 μl of DMSO were added as the negative control culture for virstatin [25].

**Culture in AKI conditions for El Tor biotype strains.** El Tor biotype strains were further analyzed in AKI conditions, as described previously [22]. Approximately 10^5^ cells/ml of the bacterial strains were inoculated in a 15 ml test tube with 10 ml of AKI broth (1.5% Bacto peptone, 0.4% yeast extract, 0.5% NaCl, 0.3% NaHCO_3_) that contained fatty acids or virstatin (at the concentrations described above) and incubated without shaking at 37°C for 4 h. The negative control cultures contained 1/100 culture volume of methanol/ethanol or 1/500 volume of DMSO. The culture was then transferred into a 250-ml flask and further cultured for 16 h with vigorous shaking [40].

### SDS-PAGE and Western Blotting

Harvested cells for western blotting of TcpA and bacterial cultures (cells and culture supernatant) for western blotting of secreted and intracellular CTB were prepared as previously described [22]. Approximately 10^7^ cells were analyzed using 15% (for CTB) and 12% (for TcpA) SDS-PAGE. Proteins separated by SDS-PAGE were transferred onto nitrocellulose membranes. The western blot analysis was performed using anti-TcpA (a generous gift from Dr. W. F. Wade, Dartmouth University, USA [48]) and anti-CTB (ab34992, Abcam, UK), with HRP-linked goat anti-rabbit IgG, (7074S, Cell Signaling, USA) used as the secondary antibody. Example western blot images and Commassie brilliant blue-stained SDS-PAGE images are shown in Fig. S1.

The western blot images were analyzed using a ChemiDoc XRS+ imaging system (Bio-Rad). The western blot band intensities representing TcpA and CTB were quantified using the ImageJ gel analysis program and ChemiDoc XRS+ Image lab. All experiments were performed three or more times, and the standard deviation is indicated by error bars.

## Results

### Monitoring CT and TCP Expression

The expression of CT and TCP was monitored by analyzing western blot images made using the whole culture (cell + culture supernatant) for CT (anti-CTB) and harvested cells for TCP (anti-TcpA), as previously described [22]. Since CT is secreted into the culture medium during culture, we analyzed the whole culture to monitor both secreted and intracellular CT. We previously showed that elevated TcpA expression is highly correlated with functional TCP production because the *V. cholerae* strains that express elevated TcpA are readily transduced by CTXΦ [24, 33, 34]. Therefore, we monitored TcpA expression as an indication of TCP formation.

Two SFAs—palmitic acid (C16, hereafter, PA) and stearic acid (C18, hereafter, SA)—and two UFAs—oleic acid (C18, hereafter, OA) and linoleic acid (C18, hereafter, LA)—were investigated to determine their effects on the transcriptional activator functions of ToxT. OA (CH_3_(CH_2_)_7_CH=CH(CH_2_)_7_COOH) and LA (CH_3_(CH_2_)_4_CH=CHCH_2_CH=CH(CH_2_)_7_COOH) differ by the number of double bonds. LA, among the UFAs, has been shown to be most efficient in inhibiting virulence gene expression in *V. cholerae*, and the SFAs have shown no effects on the transcriptional activator function of ToxT [35].

**Fig. 1:**
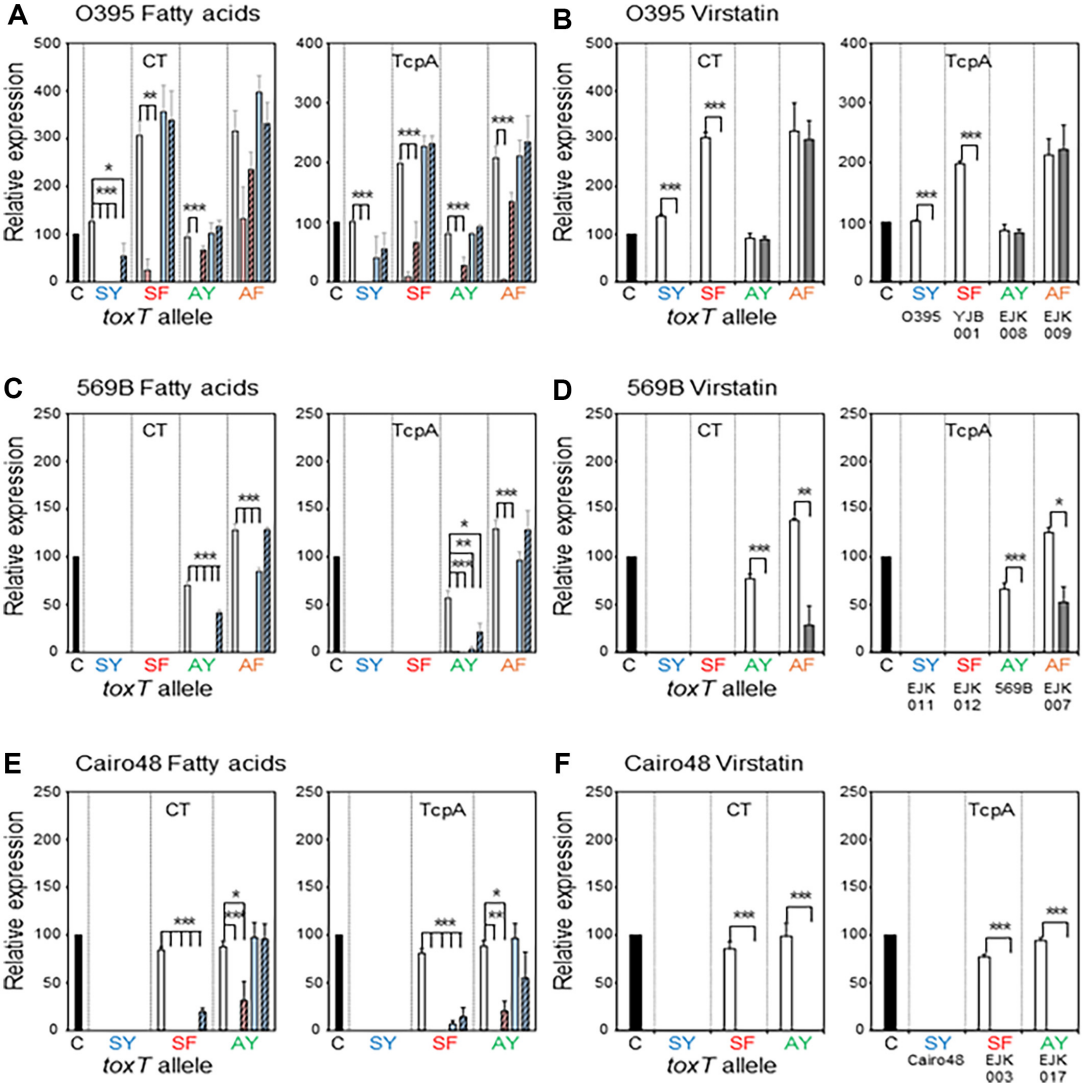
Effects of fatty acids and virstatin on the transcriptional activator functions of ToxT variants in *V. cholerae* classical biotype strains and their isogenic derivatives. The expression of CT and TcpA was monitored by western blotting. Classical biotype strains were cultured at 30°C in LB medium, and the relative expression of CT and TcpA was compared to the positive control (O395 cultured at 30°C in LB medium, pH 6.5, black bar). (**A**) Expression of CT (left) and TcpA (right) in the isogenic variants of O395 [O395 (*toxT*-SY) shown as SY, YJB001 (*toxT*-SF) shown as SF, EJK008 (*toxT*-AY) shown as AY, and EJK009 (*toxT*-AF) shown as AF] in the presence of fatty acids (methanol control: white bar, Linoleic acid: red bar, Oleic acid: striped red bar, Palmitic acid: blue bar, and Stearic acid: striped blue bar). The final concentration of the fatty acids was 0.02%. (**B**) Expression of CT (left) and TcpA (right) in the isogenic variants of O395 in the presence of 50 μM virstatin (DMSO control: white bar, and virstatin: shaded bar). (**C**) Expression of CT and TcpA in the isogenic variants of 569B in the presence of fatty acids [569B (*toxT*-AY) shown as AY, EJ007 (*toxT*-AF) shown as AF, EJK011 (*toxT*-SY) shown as SY, and EJK012 (*toxT*-SF) shown as SF] in the presence of fatty acids. (**D**) Expression of CT and TcpA in the isogenic variants of 569B in the presence of virstatin. (**E**) Expression of CT (left) and TcpA (right) in the isogenic variants of Cairo48 [Cairo48 (*toxT*-SY shown as SY, EJK003 (*toxT*-SF) shown as SF, and EJK017 (*toxT*-AY) shown as AY]in the presence of fatty acids. (**F**) Expression of CT (left) and TcpA (right) in the isogenic variants of Cairo48 in the presence of 50 μM virstatin. All experiments were performed in triplicate, and the data are expressed as the mean ± standard deviation. Statical significance was determined using one-way analysis of variance followed by the Bonferroni post hoc test to compare each mean with that of the methanol control within a group (fatty acid experiments) or using Student’s *t*-test to compare each mean with that of the DMSO control within a group (virstatin experiments). SPSS ver. 27 was used for the statistical analysis. *p ≤ 0.05, **p ≤ 0.01, and ***p ≤ 0.001.

Three classical biotype strains—O395, 569B, and Cairo48—were examined in this study. O395 and 569B are extensively studied classical biotype strains [5, 36]. The expression levels of CT and TcpA in the O395 classical biotype strain cultured in the agglutinating condition were taken as the positive control (100%), and their expression in the other strains is reported relative to it (Fig. 1). Therefore, the expression level of TcpA/CT across *V. cholerae* strains and their isogenic *toxT* variants could be directly compared.

Cairo48 and Cairo50 are included in oral cholera vaccines (OCVs). We did not investigate Cairo50 in this study because its CT and TcpA expression levels are less than 50% of the positive control in all culture conditions or under the control of any *toxT* allele [22]. In the classical biotype strains cultured in LB medium (pH not adjusted to pH 6.5), the expression of CT and TcpA in the presence of fatty acids or virstatin was monitored only at 30ºC because CT and TcpA expression at 37ºC was negligible [22].

**Fig. 2:**
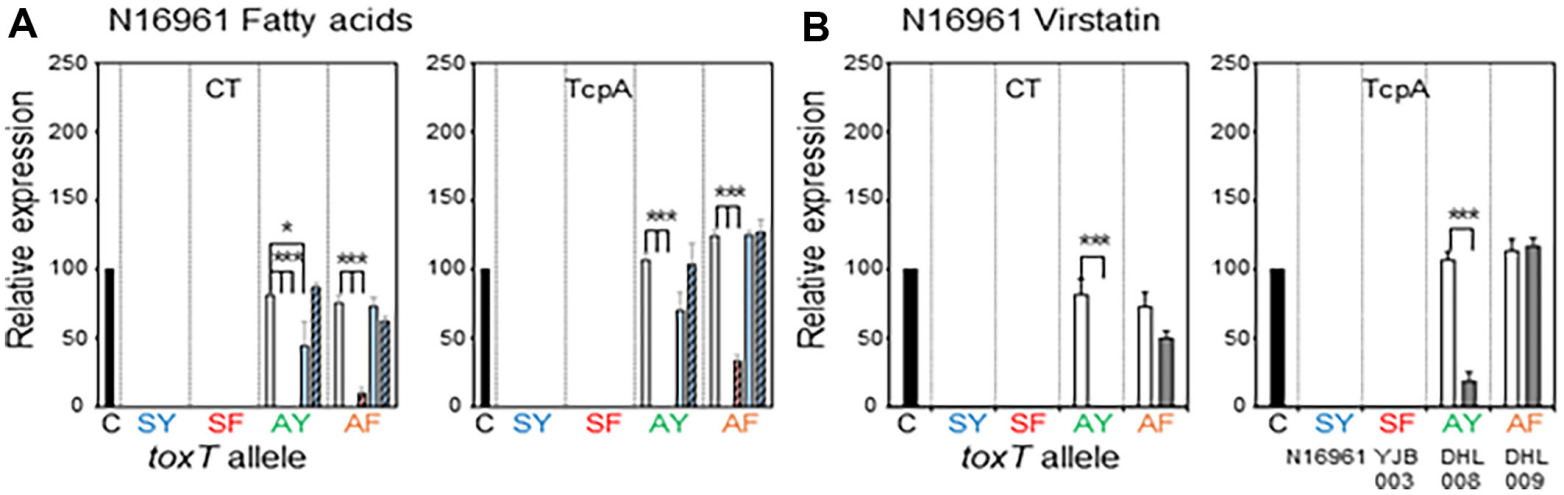
Effects of fatty acids and virstatin on the transcriptional activator functions of ToxT variants in N16961 and its isogenic derivatives. The expression of CT and TcpA was monitored by western blotting. N16961 was cultured in LB medium at 30°C and the relative expression of CT and TcpA was compared with the positive control (O395 cultured at 30°C in LB medium, pH 6.5, black bar). (**A**) Expression of CT (left) and TcpA (right) in the isogenic variants of N16961 [N16961 (*toxT*-SY) shown as SY, YJB003 (*toxT*-SF) shown as SF, DHL008 (*toxT*-AY) shown as AY, and DHL009 (*toxT*AF) shown as AF] in the presence of fatty acids (methanol control: white bar, Linoleic acid: red bar, Oleic acid: striped red bar, Palmitic acid: blue bar, and Stearic acid: striped blue bar). (B) Expression of CT (left) and TcpA (right) in the isogenic variants of N16961 in the presence of 50 μM virstatin (DMSO control: white bar, and virstatin: shaded bar). Statistical analysis was performed as described in Fig. 1.

**Fig. 3:**
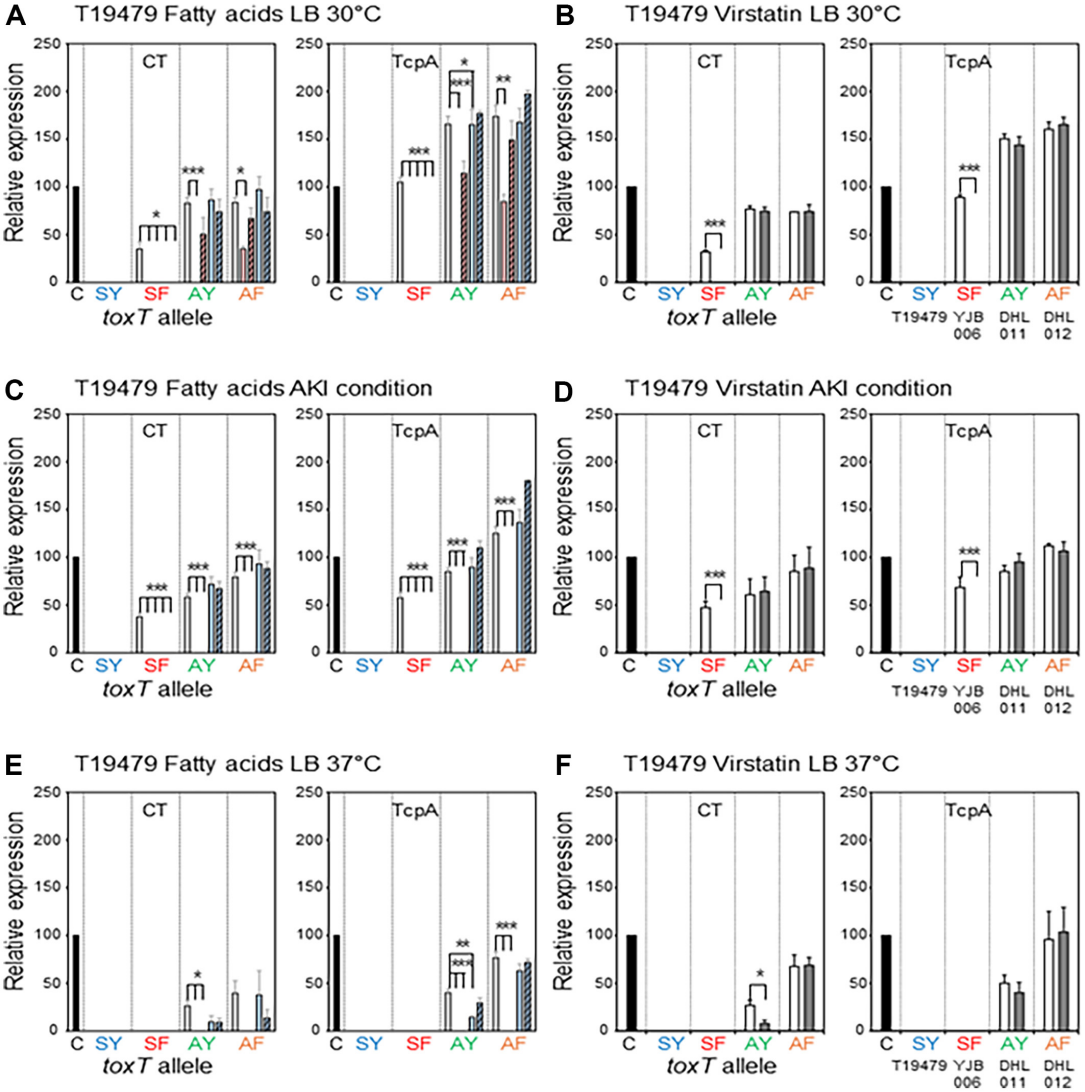
Effects of fatty acids and virstatin on the transcriptional activator functions of ToxT variants in T19479 and its isogenic derivatives. Bacteria were cultured in LB medium at 30°C (**A** and **B**), AKI conditions (**C** and **D**), and LB medium at 37°C (**E** and **F**). (**A, C**, and **E**) Expression of CT (left) and TcpA (right) in the isogenic variants of T19479 [T19479 (*toxT*-SY) shown as SY, YJB006 (*toxT*-SF) shown as SF, DHL011 (*toxT*-AY) shown as AY, and DHL012 (*toxT*-AF) shown as AF] in the presence of fatty acids (methanol control: white bar, Linoleic acid: red bar, Oleic acid: striped red bar, Palmitic acid: blue bar, and Stearic acid: striped blue bar). (**B, D**, and **F**) Expression of CT (left) and TcpA (right) in the isogenic variants of T19479 in the presence of 50 μM virstatin (DMSO control: white bar, and virstatin: shaded bar). Statistical analysis was performed as described in Fig. 1.

**Fig. 4:**
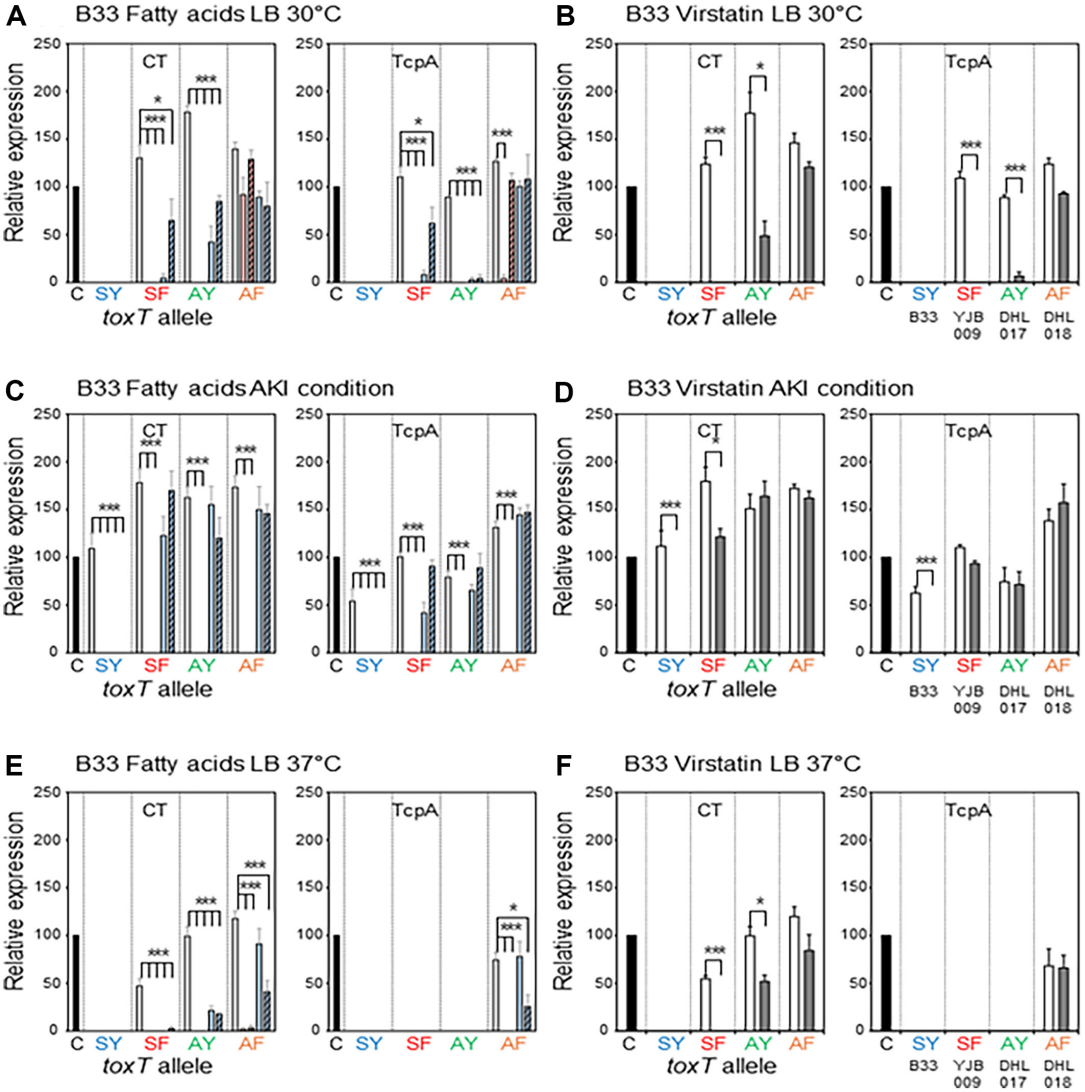
Effects of fatty acids and virstatin on the transcriptional activator functions of ToxT variants in B33 and its isogenic derivatives. Bacteria were cultured in LB medium at 30°C (**A** and **B**), AKI conditions (**C** and **D**), and LB medium at 37°C (**E** and **F**). (**A, C**, and **E**) Expression of CT (left) and TcpA (right) in the isogenic variants of B33 [B33 (*toxT*-SY) shown as SY, YJB009 (*toxT*-SF) shown as SF, DHL017 (*toxT*-AY) shown as AY, and DHL018 (*toxT*-AF) shown as AF] in the presence of fatty acids (methanol control: white bar, Linoleic acid: red bar, Oleic acid: striped red bar, Palmitic acid: blue bar, and Stearic acid: striped blue bar). (**B, D**, and **F**) Expression of CT (left) and TcpA (right) in the isogenic variants of B33 in the presence of 50 μM virstatin (DMSO control: white bar, and virstatin: shaded bar). Statistical analysis was performed as described in Fig. 1.

**Fig. 5:**
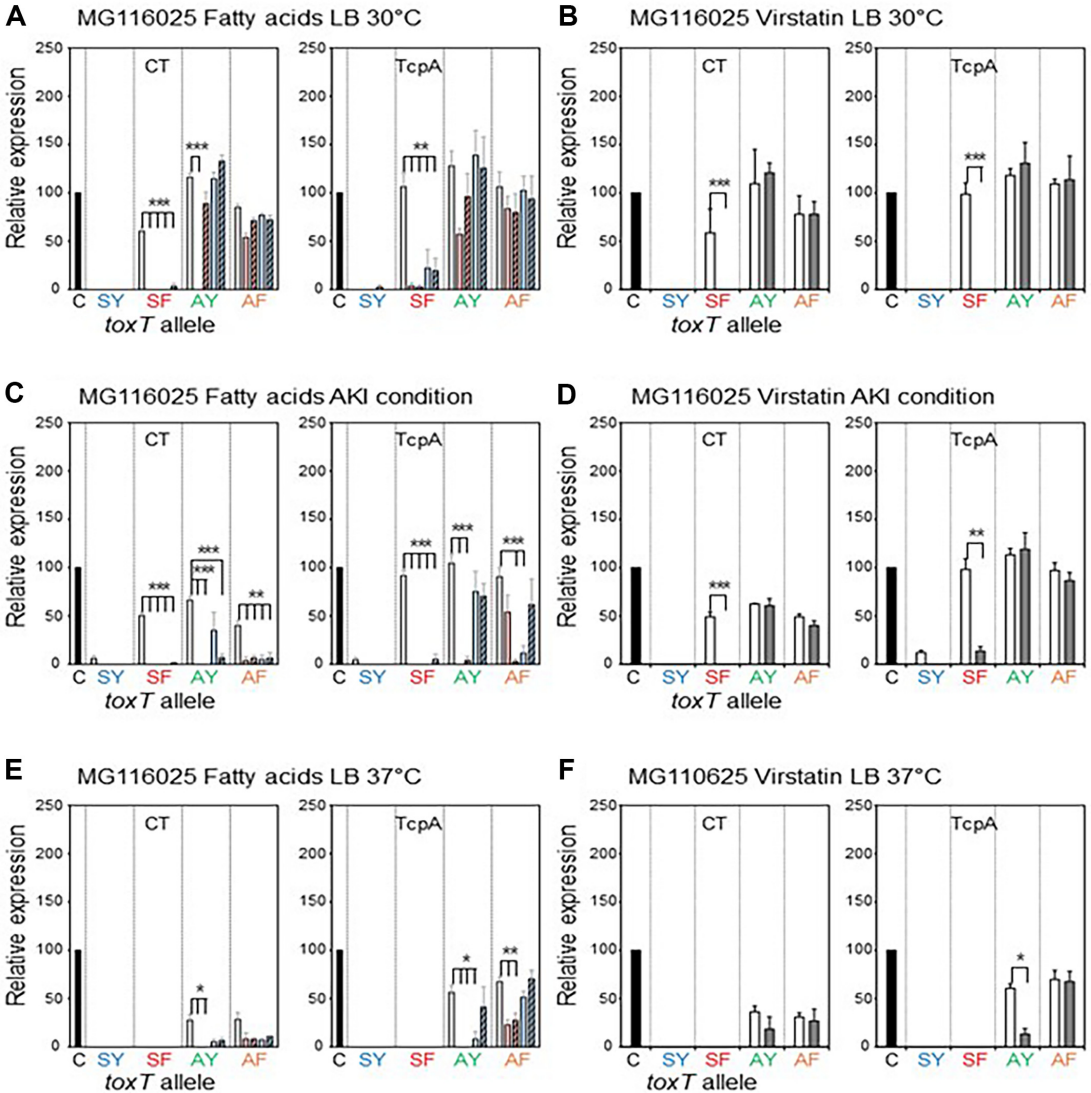
Effects of fatty acids and virstatin on the transcriptional activator functions of ToxT variants in MG116025 and its isogenic derivatives. Bacteria were cultured in LB medium at 30°C (**A** and **B**), AKI conditions (**C** and **D**), and LB medium at 37°C (**E** and **F**). (**A, C**, and **E**) Expression of CT (left) and TcpA (right) in the isogenic variants of MG116025 [MG116025 (*toxT*-SF) shown as SF, YJB015 (*toxT*-SY) shown as SY, DHL014 (*toxT*-AY) shown as AY, and DHL015 (*toxT*-AF) shown as AF] in the presence of fatty acids (methanol control: white bar, Linoleic acid: red bar, Oleic acid: striped red bar, Palmitic acid: blue bar, and Stearic acid: striped blue bar). (**B, D**, and **F**) Expression of CT (left) and TcpA (right) in the isogenic variants of MG116025 in the presence of 50 μM virstatin (DMSO control: white bar, and virstatin: shaded bar). Statistical analysis was performed as described in Fig. 1.

**Fig. 6:**
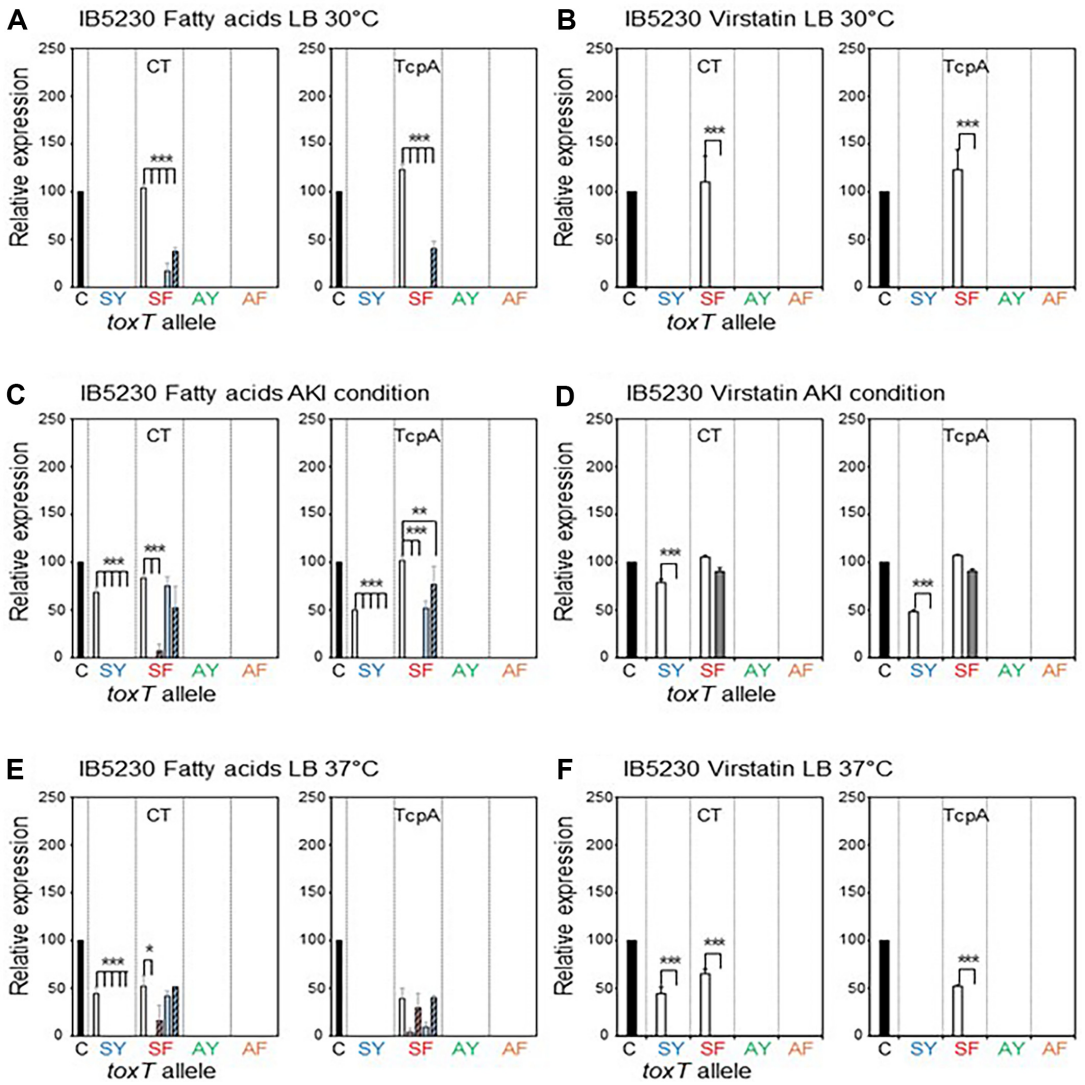
Effects of fatty acids and virstatin on the transcriptional activator functions of ToxT variants in IB5230 and its isogenic derivatives. Bacteria were cultured in LB medium at 30°C (**A** and **B**), AKI conditions (**C** and **D**), and LB medium at 37°C (**E** and **F**). (**A, C**, and **E**) Expression of CT (left) and TcpA (right) in the isogenic variants of IB5230 [IB5230 (*toxT*-SY) shown as SY, YJB020 (*toxT*-SF) shown as SF, DHL020 (*toxT*-AY) shown as AY, and DHL021 (*toxT*-AF) shown as AF] in the presence of fatty acids (methanol control: white bar, Linoleic acid: red bar, Oleic acid: striped red bar, Palmitic acid: blue bar, and Stearic acid: striped blue bar). (**B, D**, and **F**) Expression of CT (left) and TcpA (right) in the isogenic variants of IB5230 in the presence of 50 μM virstatin (DMSO control: white bar, and virstatin: shaded bar). Statistical analysis was performed as described in Fig. 1.

Five El Tor biotype strains—two Wave 1 strains, N16961 and T19479, two Wave 2 atypical El Tor strains, B33 and MG116025, and one Wave 3 atypical strain IB5230 (2010 Haitian cholera outbreak strain)—were investigated in this study [6, 37, 38]. Most El Tor biotype strains that contain the *toxT*-SY allele (Ser and Tyr at the 65^th^ and 139^th^ amino acid positions, respectively) have been shown to express CT and TCP only under AKI conditions, however, we previously showed that El Tor biotype strains can produce CT and TcpA in simple culture conditions (aerated culture in LB medium at 30ºC or 37ºC) when the *toxT*-SY allele is replaced by other *toxT* alleles (*toxT*-SF in which Tyr is replaced by Phe at the 139^th^ amino acid position, *toxT*-AY in which Ser is replaced by Ala at the 65^th^ amino acid position, and *toxT*-AF in which Ser at the 65^th^ and Tyr at 139^th^ amino acid positions are replaced by Ala and Phe, respectively) [22]. In this study, the effects of fatty acids and virstatin were investigated in El Tor biotype strains cultured in AKI conditions and LB medium at 30ºC or 37ºC (Figs. 2–6.).

### Effects of Fatty Acids and Virstatin on the Expression of Virulence Genes in Classical Biotype Strains

**O395**: UFAs were previously shown to inhibit the expression of virulence genes in strain O395 (*toxT*-SY), whereas SFAs (especially SA) had no effects [35]. In this study, UFAs inhibited the virulence gene expression of O395, as anticipated (Fig. 1A); however, the SFAs also inhibited virulence gene expression (especially CTB) in O395 (Fig. 1A).

The expression of virulence genes in YJB001 (an isogenic derivative of O395 in which *toxT*-SY is replaced by *toxT*-SF) was also inhibited by the UFAs, but the SFAs showed no inhibitory effects on expression in YJB001 (Fig. 1A).

The two UFAs showed different effects on virulence gene expression in EJK008 (an isogenic derivative of O395 in which the *toxT*-SY allele is replaced by *toxT*-AY). LA entirely inhibited the expression of CT and TcpA in EJK009 (*toxT*-AF), but the inhibitory effects of PA were far less than those of LA. The SFAs did not affect the expression of virulence genes in EJK008 (*toxT*-AY) (Fig. 1A).

EJK009 (*toxT*-AF) expresses an enhanced amount of CT (approximately 300% higher than the positive control) and TcpA (200% higher than the positive control). LA inhibited expression of TcpA entirely; however, the inhibitory effects of LA on CT expression were low, resulting in a CT expression level similar to the positive control (Fig. 1A). OA and the SFAs did not show inhibitory effects in EJK009.

Virstatin entirely inhibited virulence gene expression in O395 (*toxT*-SY) and YJB001 (*toxT*-SF). However, the virulence gene expression in EJK008 (*toxT*-AY) and EJK009 (*toxT*-AF) was not affected by virstatin (Fig. 1B).

**569B:** The classical biotype 569B strain originally contains the *toxT*-AY allele [22] and its virulence gene expression is comparable to the positive control (Fig. 1C and 1D). Both the UFAs and SFAs (especially PA) inhibited the virulence gene expression of 569B (*toxT*-AY) (Fig. 1C).

Virulence gene expression was enhanced up to approximately 150% in EJK007 (an isogenic derivative of 569B in which the *toxT*-AY allele was replaced by *toxT*-AF). The UFAs efficiently inhibited the expression of CT and TcpA in EJK007 (*toxT*-AF) (Fig. 1C). The expression of CTB in EJK007 (*toxT*-AF) was slightly inhibited by PA, but the remaining expression was still as much as the positive control (Fig. 1C). SA did not interfere with the expression of virulence genes in EJK007 (*toxT*-AF).

Virstatin efficiently inhibited virulence gene expression in 569B; however, its inhibitory effects in EJK007 (*toxT*-AF) were low. Specifically, the residual expression of TcpA was 50% of the positive control (Fig. 1D).

No virulence genes were expressed by EJK011 (*toxT*-SY) or EJK012 (*toxT*-SF), and neither the fatty acids nor virstatin produced any effects (Fig. 1D).

**Cairo48:** Cairo48 originally contains the *toxT*-SY allele and it does not express virulence genes in the agglutinating condition nor LB medium, unlike the other classical biotype strains [22]. EJK003 (*toxT*-SF variant) and EJK017 (*toxT*-AY variant) showed CT and TcpA expression levels similar to the positive control. We could not construct the isogenic variant containing the *toxT*-AF allele [22].

Both the UFAs and SFAs had inhibitory effects on virulence gene expression in EJK003 (*toxT*-SF), but the SFAs did not show inhibitory effects on EJK017 (*toxT*-AY) (Fig. 1E). Virstatin completely inhibited virulence gene expression in EJK003 (*toxT*-SF) and EJK017 (*toxT*-AY) (Fig. 1F).

### Effects of Fatty Acids and Virstatin on the Expression of Virulence Genes in El Tor Biotype Strains

**N16961:** Neither N16961 (*toxT*-SY allele) nor its isogenic derivative YJB003 (*toxT*-SF) expressed virulence genes in LB medium or AKI conditions [21, 22]. DHL008 (*toxT*-AY) and DHL009 (*toxT*-AF) expressed as much CT and TcpA as the positive control only when cultured in LB medium at 30ºC. The virulence gene expression of DHL008 (*toxT*-AY) was entirely inhibited by the UFAs (Fig. 2A). Among the SFAs, PA inhibited CTB expression but not TcpA (Fig. 2A), and SA did not show any inhibitory effects on virulence gene expression in DHL008 (*toxT*-AY). CTB and TcpA expression in DHL009 (*toxT*-AF) was inhibited by UFAs, although the inhibitory effects of OA were slightly diminished (Fig. 2A). Virulence gene expression in DHL009 (*toxT*-AF) was not affected by the SFAs (Fig. 2A).

Virstatin inhibited virulence gene expression in DHL008 (*toxT*-AY); however, it did not affect virulence gene expression in DHL009 (*toxT*-AF) (Fig. 2B).

**T19479:** Virulence genes were not expressed by T19479 (*toxT*-SY) at any culture conditions, but the isogenic derivatives YJB006 (*toxT*-SF), DHL011 (*toxT*-AY), and DHL012 (*toxT*-AF) showed virulence gene expression in LB medium (30ºC and 37ºC) and AKI conditions [22], except for YJB006 (*toxT*-SF) cultured in LB medium at 37ºC (Figs. 3 and S1). Virulence gene expression in YJB006 (*toxT*-SF) cultured in LB medium at 30ºC and AKI conditions was entirely inhibited by both the UFAs and SFAs (Fig. 3A and 3C).

Among the UFAs, LA inhibited virulence gene expression in DHL011 (*toxT*-AY) cultured in LB medium (30ºC), but the effects of OA were not significant (Fig. 3A). However, both UFAs efficiently inhibited virulence gene expression in DHL011 (*toxT*-AY) cultured in AKI conditions. The expression of CT and TcpA in DHL011 (*toxT*-AY) cultured in LB medium at 30°C or AKI conditions was not influenced by the SFAs (Fig. 3C), though the TcpA expression in LB medium at 37°C was slightly inhibited by PA (Fig. 3E). However, virulence gene expression in DHL011 (*toxT*-AY) cultured in LB medium at 37°C was lower than 50% of the positive control; therefore, the effects of fatty acids in this condition might not be significant.

Virulence gene expression in DHL012 (*toxT*-AF) cultured in LB medium at 30°C was slightly inhibited by LA; however, a considerable amount of CT and TcpA was still expressed (Fig. 3A). Virulence gene expression in DHL012 (*toxT*-AF) was not influenced by OA or the SFAs (Fig. 3A). The UFAs efficiently inhibited virulence gene expression in DHL012 (*toxT*-AF) cultured in AKI conditions (Fig. 3C). The expression of TcpA in DHL012 (*toxT*-AF) cultured in LB medium at 37°C was inhibited by the UFAs and unaffected by the SFAs (Fig. 3E).

Virstatin inhibited virulence gene expression in YJB006 (*toxT*-SF) cultured in LB medium at 30°C and AKI conditions. However, virulence gene expression in DHL011 (*toxT*-AY) and DHL012 (*toxT*-AF) cultured in any conditions was not affected by virstatin, except for DHL011 (*toxT*-AY) in LB medium at 37°C (Fig. 3B, 3D, and 3E).

**B33:** B33 (originally containing the *toxT*-SY allele) is a Wave 2 atypical El Tor strain that expresses virulence genes when cultured in AKI conditions, but not when cultured in LB medium (Fig. 4) [22]. Its expression of CT and TcpA in AKI conditions was completely inhibited by both the UFAs and SFAs (Fig. 4C).

Replacing the *toxT*-SY allele of B33 with other *toxT* alleles—YJB009 (*toxT*-SF), DHL017 (*toxT*-AY), and DHL018 (*toxT*-AF)—enabled this strain to express virulence genes in both AKI conditions and LB medium at 30°C and 37°C (Fig. 4A, 4C, and 4E).

Virulence gene expression in YJB009 (*toxT*-SF) cultured in LB medium at 30°C or AKI conditions was entirely inhibited by the UFAs (Fig. 4A and 4C). PA also inhibited virulence gene expression in YJB009 (*toxT*-SF) cultured in LB medium at 30°C; however, its inhibitory effects were marginal in AKI conditions (Fig. 4C). SA significantly inhibited virulence gene expression in YJB009 (*toxT*-SF) in LB medium at 30°C; however, the expression of CT and TcpA was still more than 50% of the positive control (Fig. 4A). In AKI conditions, the SFAs showed no inhibitory effects on the virulence gene expression of YJB009 (*toxT*-SF), although PA slightly reduced TcpA expression (Fig. 4C).

The UFAs entirely inhibited virulence gene expression in DHL017 (*toxT*-AY) in all culture conditions; however, the SFAs showed discrete effects depending on the culture condition (Fig. 4A, 4C, and 4E). The expression of CTB was significantly inhibited by the SFAs, and TcpA expression was entirely inhibited when DHL017 (*toxT*-AY) was cultured in LB medium at 30°C (Fig. 4A). However, virulence gene expression was not affected by the SFAs when DHL017 (*toxT*-AY) was cultured under AKI conditions (Fig. 4C). CT expression in DHL017 (*toxT*-AY) cultured in LB medium at 37°C was inhibited by the SFAs (Fig. 4E).

CT expression in DHL018 (*toxT*-AF) cultured in LB medium at 30°C was not inhibited by the UFAs or SFAs, and TcpA expression was inhibited only by LA (Fig. 4A). When DHL018 (*toxT*-AF) was cultured under AKI conditions, virulence gene expression was entirely inhibited by the UFAs and unaffected by the SFAs. When DHL018 (*toxT*-AF) was cultured in LB medium at 37°C, virulence gene expression was inhibited by SA (Fig. 4E).

Virstatin efficiently inhibited the virulence gene expression of B33 (*toxT*-SY) cultured under AKI conditions (Fig. 4D). Virstatin also inhibited virulence gene expression in YJB009 (*toxT*-SF) cultured in LB medium (30°C and 37°C); however, its inhibitory effects vanished when YJB009 (*toxT*-SF) was cultured under AKI conditions (Fig. 4B, 4D, and 4F). Similarly, the virulence gene expression of DHL017 (*toxT*-AY) cultured in LB medium at 30°C was significantly reduced by virstatin, but no inhibitory effects were observed when DHL017 (*toxT*-AY) was cultured in AKI conditions. The virulence gene expression of DHL018 (*toxT*-AF) was not affected by virstatin in any culture conditions (Fig. 4B, D, and 4F).

**MG110625:** MG116025 originally contains *toxT*-SF and expresses CT and TcpA when cultured in AKI conditions and LB medium at 30°C [22, 23, 34]. The virulence gene expression of MG116025 (*toxT*-SF) was entirely inhibited by both the UFAs and SFAs under AKI conditions and when cultured in LB medium at 30°C (Fig. 5A, 5C, and 5E). An isogenic derivative that contained *toxT*-SY—YJB015—did not express virulence genes under any culture conditions.

The CTB expression of DHL014 (*toxT*-AY) cultured in LB medium at 30°C was inhibited by LA, but TcpA expression was only marginally reduced by LA (Fig. 5A).

Neither OA nor the two SFAs inhibited virulence gene expression of DHL014 (*toxT*-AY) cultured in LB medium at 30°C. The UFAs inhibited the virulence gene expression of DHL014 (*toxT*-AY) cultured in AKI conditions; however, the inhibition of CTB expression by PA and TcpA expression by SA were marginal (Fig. 5C). The CTB expression of DHL014 (*toxT*-AY) and DHL015 (*toxT*-AF) in LB medium at 37ºC was negligible, and TcpA expression was inhibited by the UFAs and PA (Fig. 5E).

Virstatin efficiently inhibited the virulence gene expression of MG116025 cultured in AKI conditions and LB medium at 30°C (Fig. 5B and 5D). However, virstatin showed no inhibitory effects on the expression of virulence genes in DHL014 (*toxT*-AY) and DHL015 (*toxT*-AF) under any culture conditions, except for the TcpA expression of DHL015 (*toxT*-AF) cultured in LB medium at 37°C (Fig. 5F).

**IB5230:** IB5230 is the hypervirulent 2010 Haiti cholera outbreak strain [38, 39]. It originally contains the *toxT*-SY allele and expresses virulence genes under AKI conditions. Only CT was expressed when it was cultured in LB medium at 37°C (Fig. 6). The virulence gene expression of IB5230 (*toxT*-SY) under those conditions was entirely inhibited by both the UFAs and SFAs (Fig. 6C and 6E).

Virulence gene expression was stimulated in LB medium at 30°C when the *toxT*-SY allele was replaced with the *toxT*-SF allele (YJB020), and both the UFAs and SFAs significantly inhibited virulence gene expression in YJB020 (*toxT*-SF) cultured in LB medium at 30°C (Fig. 6A). The inhibition of virulence gene expression by the SFAs was negligible under AKI conditions except for TcpA expression in the presence of PA, which was slightly reduced (Fig. 6C). LA inhibited the CTB expression of YJB020 (*toxT*-SF) cultured in LB medium at 37°C, whereas OA and the SFAs showed negligible effects on virulence gene expression in those conditions (Fig. 6E). Replacing the *toxT*-SY allele with either *toxT*-AY (DHL020) or *toxT*-AF (DHL021) entirely abolished virulence gene expression.

The virulence gene expression of IB5230 (*toxT*-SY) was inhibited by virstatin under AKI conditions (Fig. 6D). Intriguingly, the virulence gene expression of YJB020 (*toxT*-SF) in LB medium was inhibited by virstatin; however, it was not affected by virstatin under AKI conditions (Fig. 6B, 6D, and 6F).

## Discussion

The expression of virulence genes in laboratory culture conditions is indispensable in studying the pathogenesis of infectious diseases, and *V. cholerae* strains do not express virulence genes under simple laboratory culture conditions [40]. Virulence gene expression in *V. cholerae* is tightly regulated by the ToxR regulon [10]. ToxT, which is produced through the ToxR regulon, has been shown to directly activate transcription of virulence genes, including *ctxAB* and *tcpA*, in *V. cholerae* [10].

Three *toxT* alleles have been identified among clinical isolates of *V. cholerae*—*toxT*-SY in most *V. cholerae* isolates, *toxT*-SF in the MG116025 Wave 2 atypical El Tor strain, and *toxT*-AY in the 569B classical biotype strain [22]. The *toxT*-SF allele in MG116025 was identified because it can be readily transduced by the classical type CTXΦ (CTXΦ^cla^), which suggested that it produced functional TCP as the receptor for CTXΦ [33, 34]. When introduced into *V. cholerae* strains other than MG116025, the *toxT*-SF allele enhanced the virulence gene expression of some classical biotype strains and induced virulence gene expression in El Tor biotype strains in simple laboratory culture conditions (shake culture in LB medium), that do not usually induce virulence gene expression [21, 34].

We have shown that the transcriptional activator functions of variant *toxT* alleles, including the artificially generated *toxT*-AF, differ by strain and culture conditions; in other words, there are no universal virulence gene-inducing conditions or *toxT* alleles [22]. For example, the *toxT*-AY and *toxT*-AF alleles generally activate the expression of CT and TcpA in many *V. cholerae* strains in various culture conditions, but in IB5230 (a Wave 3 atypical El Tor strain), virulence gene expression was completely abolished when the *toxT*-AY or *toxT*-AF allele was introduced [22]. We are investigating potential molecular mechanisms of characteristics of the different *toxT* alleles in *V. cholerae* strains, including alterations in molecular structure, interaction with inhibitors, and differential expression or removal of ToxT.

WASH (water, sanitation, and hygiene) is considered to be an effective preventive intervention against cholera, and antibiotics and OCVs are commonly used to control cholera [41, 42]. While more efficient vaccine regimens and improved vaccine strains are under development [43, 44], various methods of inhibiting the expression of virulence genes in *V. cholerae* have been suggested to counter the increase in antibiotics resistance and improve the moderate efficacy of OCVs [45, 46].

Fatty acids—especially UFAs—have been investigated as agents for inhibiting virulence gene expression in *V. cholerae* [30, 32]. In addition, small-molecule inhibitors targeting ToxT are considered to be good alternatives to antibiotics [25-27]. Virstatin, which was developed as a small-molecule inhibitor of ToxT, and UFAs have been shown to inhibit the dimerization and DNA binding of ToxT, respectively, thereby reducing the expression of virulence genes in *V. cholerae*.

In this report, we monitored the effectiveness of fatty acids (saturated and unsaturated) and virstatin in inhibiting the transcriptional activator functions of four *toxT* alleles in eight *V. cholerae* strains because each *toxT* allele showed discrete transcriptional activator functions in each strain and culture condition [22].

Overall, the fatty acids and virstatin inhibited the expression of virulence genes in *V. cholerae*; however, the effectiveness varied from strong to nonexistent depending on the *toxT* allele and culture conditions. In all strains, neither the fatty acids nor virstatin showed any stimulatory effects on virulence gene expression in strains under the control of particular *toxT* alleles or in culture conditions, in which the virulence genes were not expressed.

The inhibitory effects of the fatty acids and virstatin were reduced when virulence gene expression was enhanced by the culture conditions or use of a particular *toxT* allele. For example, the enhanced expression of virulence genes seen in some strains with a specific *toxT* allele in particular culture conditions, such as strain O395 with the *toxT*-AF allele at 30°C, could be inhibited by UFAs by certain extent [22], but the remaining expression was still higher than the positive control. Thus, the ability of fatty acids or virstatin in controlling virulence gene expression in *V. cholerae* might be insufficient.

In summary, the inhibition of the transcriptional activator functions of ToxT by fatty acids and virstatin varies by *toxT* allele, *V. cholerae* strain, and culture conditions, which implies that *V. cholerae* strains can avoid the anti-virulence effects of fatty acids and small-molecule inhibitors of ToxT by acquiring variant *toxT* alleles. Therefore, various *toxT* alleles in several *V. cholerae* strains should be considered when developing small-molecule inhibitors of ToxT to find candidate molecules that can generally inhibit the activity of various ToxT alleles.

## Supplemental Materials

Supplementary data for this paper are available on-line only at http://jmb.or.kr.



## Figures and Tables

**Table 1:** Bacterial strain list.

Strains	*toxT* genotypes	Genome information and references	Strains	*toxT* genotypes	Genome information and references
Classical biotype El Tor biotype					
O395	O395-*toxT*-SY (65S, 139Y)	CP000626/CP000627 [5]	N16961	N16961-*toxT*-SY	AE003852/AE003853 [49]
YJB001	O395-*toxT*-SF (65S, 139F)	[21]	YJB003	N16961-*toxT*-SF	[21]
EJK008	O395-*toxT*-AY (65A, 139Y)	[22]	DHL008	N16961-*toxT*-AY	[22]
EJK009	O395-*toxT*-AF (65A, 139F)	[22]	DHL009	N16961-*toxT*-AF	[22]
569B	569B-*toxT*-AY	DADXPZ0100 00000	T19479	T19479-*toxT*-SY	ERS013250 [5]
EJK007	569B-*toxT*-AF	[24]	YJB006	T19479-*toxT*-SF	[21]
EJK011	569B-*toxT*-SY	[22]	DHL011	T19479-*toxT*-AY	[22]
EJK012	569B-*toxT*-SF	[22]	DHL012	T19479-*toxT*-AF	[22]
Cairo48	Cairo48-*toxT*-SY	ERS013171 [5]	B33	B33-*toxT*-SY	ACHZ00000000 [50]
EJK003	Cairo48-*toxT*-SF	[24]	YJB009	B33-*toxT*-SF	[21]
EJK017	Cairo48-*toxT*-AY	[22]	DHL017	B33-*toxT*-AY	[22]
			DHL018	B33-*toxT*-AF	[22]
			MG116025	MG116025-*toxT*-SF	ERS013135 [5]
			YJB015	MG116025-*toxT*-SY	[21]
			DHL014	MG116025-*toxT*-AY	[22]
			DHL015	MG116025-*toxT*-AF	[22]
			IB5230	IB5230-*toxT*-SY	AELH00000000.1[38]
			YJB020	IB5230-*toxT*-SF	[21]
			DHL020	IB5230-*toxT*-AY	[22]
			DHL021	IB5230-*toxT*-AF	[22]
