# Supplementary figures and images for: Variations in the Antivirulence Effects of Fatty Acids and Virstatin against Vibrio cholerae Strains
Source: J Microbiol Biotechnol. 2024 Jul 19;34(9):1757–68. doi: 10.4014/jmb.2405.05002 (PMC11485679; doi:10.4014/jmb.2405.05002)

Fig.S1. T19479

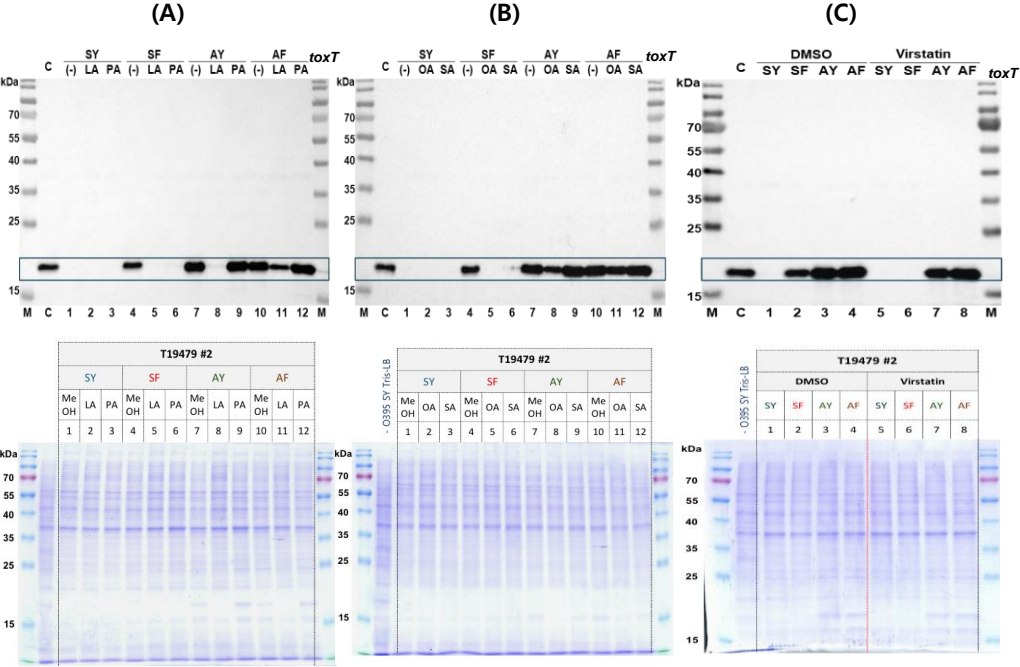

Supplement: Supplementary file 1 [file jmb-34-9-1757-supple.pdf]
